# A Review on Recent Advancement on Age-Related Hearing Loss: The Applications of Nanotechnology, Drug Pharmacology, and Biotechnology

**DOI:** 10.3390/pharmaceutics13071041

**Published:** 2021-07-08

**Authors:** Jacqueline Chester, Edan Johnston, Daniel Walker, Melissa Jones, Corina Mihaela Ionescu, Susbin Raj Wagle, Božica Kovacevic, Daniel Brown, Momir Mikov, Armin Mooranian, Hani Al-Salami

**Affiliations:** 1 The Biotechnology and Drug Development Research Laboratory, Curtin Medical School & Curtin Health Innovation Research Institute, Curtin University, Perth, WA 6102, Australia; j.chester@graduate.curtin.edu.au (J.C.); edan.johnston@student.curtin.edu.au (E.J.); daniel.walker1@postgrad.curtin.edu.au (D.W.); melissa.a.jones@postgrad.curtin.edu.au (M.J.); c.ionescu@postgrad.curtin.edu.au (C.M.I.); susbinraj.wagle@postgrad.curtin.edu.au (S.R.W.); bozica.kovacevic@postgrad.curtin.edu.au (B.K.); daniel.brown2@curtin.edu.au (D.B.); 2 Hearing Therapeutics, Ear Science Institute Australia, Queen Elizabeth II Medical Centre, Perth, WA 6102, Australia; 3 Department of Pharmacology, Toxicology and Clinical Pharmacology, Faculty of Medicine, University of Novi Sad, Hajduk Veljkova 3, 21000 Novi Sad, Serbia; MOMIR.MIKOV@mf.uns.ac.rs

**Keywords:** antioxidant, age-related hearing loss, age, metformin, biguanides, bile acids

## Abstract

Aging is considered a contributing factor to many diseases such as cardiovascular disease, Alzheimer’s disease, and hearing loss. Age-related hearing loss, also termed presbycusis, is one of the most common sensory impairments worldwide, affecting one in five people over 50 years of age, and this prevalence is growing annually. Associations have emerged between presbycusis and detrimental health outcomes, including social isolation and mental health. It remains largely untreatable apart from hearing aids, and with no globally established prevention strategies in the clinical setting. Hence, this review aims to explore the pathophysiology of presbycusis and potential therapies, based on a recent advancement in bile acid-based bio-nanotechnologies. A comprehensive online search was carried out using the following keywords: presbycusis, drugs, hearing loss, bile acids, nanotechnology, and more than 150 publications were considered directly relevant. Evidence of the multifaceted oxidative stress and chronic inflammation involvement in cellular damage and apoptosis that is associated with a loss of hair cells, damaged and inflamed stria vascularis, and neuronal signalling loss and apoptosis continues to emerge. New robust and effective therapies require drug delivery deeper into the various layers of the cochlea. Bile acid-based nanotechnology has gained wide interest in its permeation-enhancing ability and potential for numerous applications in treating presbycusis.

## 1. Introduction

Worldwide, hearing loss effects an estimated 1.57 billion people and is the third largest contributor of years lived with disability as of 2019 and this figure is predicted to increase annually [1]. Sensorineural hearing loss (SNHL) refers to the loss of hearing via maladies of the inner ear, most commonly caused by irreversible damage and the subsequent loss of cochlea hair cells and auditory neurons. A specific category of SNHL is age-related hearing loss (ARHL), more commonly termed presbycusis. The emergence of links between an excess of reactive oxygen species (ROS) and SNHL-associated damage has spurred preventative and restorative pharmacological management research for SNHL and specifically ARHL. Even with this newfound focus into antioxidant therapy for ARHL, there is still a current unmet need in the pharmacological prevention, management, and reversal of hearing loss [2].

Bile acids have previously been utilised to improve pharmacokinetics of drugs with poor delivery profiles [3]. In addition to examining the recent developments in ARHL pathophysiology and potential drug therapies, this review will explore the benefits of bile acid incorporation into drug delivery models in the context of ARHL.

## 2. Methodology

A literature search was conducted using Endnote X9 online search, PubMed and Google Scholar incorporating the following key words: “presbycusis”, “age-related hearing loss”, “oxidative stress”, “aging”, “antioxidant”, “biotechnology”, “bile acids, “nanotechnology”, and “bile acids”. Studies from 2017 to 2020 in the English language were considered for potential use. Abstracts were read for studies involved and those using a combination of the previously mentioned terms were considered and read in full. All studies sourced externally from these search criteria were found via the citation list of established references.

## 3. Function of the Ear

The human ear is comprised of the following three sections: the outer ear, middle ear, and inner ear. The external and visible structure of the ear is the auricle, which is the most lateral part of the outer ear and has acoustically engineered anatomy functioning to improve sound localisation and facilitate the amplification and subsequent channelling of sound. This structure funnels into the external auditory canal and the tympanic membrane, terminating at the tympanic membrane. Filled with air, the middle ear compartment houses the Eustachian tube, muscles of the middle ear, structures of innervation, and the ossicles. The Eustachian tube equalises the pressure within the middle ear to the ambient pressure of the outer ear via its connective opening in the nasopharynx, allowing for the optimal functionality of the tympanic membrane. The ossicles consist of three interconnected bones known as the malleus (fixed to the tympanic membrane), the stapes (fixed to the oval window to the inner ear), and the incus (connecting the malleus and stapes) [4]. Attached to the stapes is the stapedius muscle and attached to the malleus is the tensor tympani muscle, which are innervated by the facial and trigeminal cranial nerves, respectively. In response to acoustic trauma below a frequency of 2 kHz, these muscles contract, reducing ossicular auditory amplification via the stabilisation of the bony chain [5]. This apparatus delivers and amplifies vibrational movement from the external ear, through the middle ear, and to the cochlea [4].

The inner ear encloses the cochlea, vestibular apparatus, and the semi-circular canals [4]. The cochlea is spiral-like in shape and compartmentalised into the following three parallel membranous chambers: the endolymph-containing scala media and the scala vestibuli and the scala tympani, both containing perilymph and travelling above and below the scala media, respectively [6]. Perilymph is high in Na^+^ concentration and low in K^+^ concentration with a net ionic effect of −80 mV [7]. Conversely, the endolymph within the scala media has a concentration of high K^+^ and low Na^+^ that is primarily maintained by the Na^+^-K^+^-ATPase pumps on the apical border of the stria vascularis epithelial cells forming the lateral boundary of the scala media. The scala media is bordered by the Reissner’s membrane and the basilar membrane, segregating it from the scala vestibuli and the scala tympani, respectively [8]. Within the centre of the cochlea structure is the modiolus containing the spiral ganglions, which project and combine to form the cochlear nerve referring action potentials from the organ of Corti sensory cells to the brain for sound recognition and processing [9].

The organ of Corti is considered the functional unit of auditory processing within the cochlea for, and is made up of, hair cells and their supporting cells arranged in a highly organised asymmetrical mosaic pattern along the span of the basilar membrane. Hair cells are considered the sensory cells of the cochlea, categorised by their location and function. Outer hair cells (OHCs) are in a triad row along the basilar membrane, and they function to amplify specific frequencies of vibrational movement. Inner hair cells (IHCs) travel in a singular row along the basilar membrane and are responsible for the transduction of vibrational movement from the cochlear to the auditory neurons. The supporting cells are organised in very precise patterns within the organ of Corti, including, but not limited to, pillar cells, Hensen’s cells, phalangeal cells, and Dieter’s cells [6].

When sound waves enter the external ear and are transferred to the oval window via the ossicular chain, the scala vestibuli is mobilised. This movement travels the length of the cochlea and returns to the base of the scala tympani, displacing the round window. This induces a wave of movement along the basilar membrane to be absorbed and detected by the correlating OHCs. Stereocilia atop OHCs are connected to adjacent stereocilia K^+^ ion channel gates via tip-links. The shear stress of the wave motion pulls the tip-links, opening the K^+^ ion channels allowing cellular influx of endolymph K^+^, depolarising the OHCs [10]. A depolarised OHC rhythmically contracts, actively amplifying the mechanical stimulation of an IHC, which depolarises via the same mechanism. The depolarisation of an IHC triggers the influx of Ca^2+^, triggering the release of glutamate into the synapse preceding the afferent neuron connection of a spiral ganglion. This signal is then projected by the spiral ganglion to the brain via the vestibulocochlear nerve [9,11].

## 4. Age-Related Hearing Loss

Age-related hearing loss is a disease that is currently irreversible, resulting from the cumulative effects of long-term damage to structures of the inner ear and associated neurons. Currently, ARHL is the largest contributing morbidity towards years lived with disability for populations over 70 years of age [1]. It was estimated that, in 2019, the global economic cost of hearing loss was estimated to be over $981 billion (2019 purchasing power parity adjusted international dollars) and 30.7% of that cost was attributed to health care in adult hearing loss [12]. The presentation of ARHL begins typically with difficulty discerning speech, particularly in noisy environments, then progresses to the loss of hearing sensitivity, a reduced ability to localise sound origin, and a decrease in central auditory processing [13,14]. The risk of cognitive decline, depression, and social isolation is increased for those with ARHL, and over the past decade, ARHL has also been associated with dementia development [14,15,16,17,18,19,20]. Treatment of symptoms is limited to hearing-aids and, in more severe cases, cochlear implants; but currently, no pharmacological intervention exists. The increasing health and economic burdens associated with ARHL demonstrates the need for a suitable therapeutic intervention [1,12].

### 4.1. Pathophysiology of Age-Related Hearing Loss

The definition of aging is controversial and highly dependent on the context in which it is approached, whether it is physiological, molecular, psychological, or functional. In summary, it is a time-dependent process denoting the cumulative loss of function and increase in vulnerability [21]. In 1956, the free radical theory of aging was established by Denham Harman, attributing aging adjacent functional loss as a consequence of structural damage to macromolecules via oxidative stress [22]. This theory was updated in 2014 to the “free radical theory of frailty” to highlight the relationship of oxidative stress with the state of frailty, as opposed to its previously accepted relationship with chronological age [23]. The irreversible loss of hair cells and spiral ganglion cells, and degeneration of the stria vascularis is believed to be the cause of ARHL [14]. The exact mechanisms behind damage to these areas is still being explored, but over time more evidence has come to light supporting the theory that oxidative stress and inflammation incur the deleterious effects leading to this damage [24,25].

#### 4.1.1. Oxidative Stress

Oxidative stress is the unequillibrium in pro-oxidant production against antioxidant production and buffering capacity in favour of pro-oxidants. Pro-oxidant compounds are primarily reactive oxygen species (ROS) capable of exchanging one of their free valence electrons with oxygen in the body incurring damage of various macromolecules, but predominantly, nucleic acids, lipids, and proteins [26,27]. Several factors increase ROS production in ARHL, such as noise-exposure, ototoxic drugs, and age-related devascularisation and blood flow reduction [14,28,29]. In addition, antioxidant activity is concurrently reduced with age, exacerbating the ROS-antioxidant imbalance [2]. Mitochondria produce the majority of ROS as by-products of cellular respiration, specifically from the Krebs’s cycle and oxidative phosphorylation process [29]. Excessive ROS production adversely affects mitochondrial DNA (mtDNA), incurring deletions and mutations within the genome. Alterations in mtDNA leads to extreme dysregulation in oxidative phosphorylation, further increasing ROS production and inducing cellular damage capable of initiating apoptotic pathways [30,31].

Numerous studies have clarified the involvement of the Kelch-like ECH-associated protein 1-NF-E2-related factor 2 (NRF2-KEAP1). Under normal functioning conditions, NRF2 is inhibited by KEAP-1, but in conditions of increased ROS, KEAP-1 is modified, liberating NRF2 to initiate robust antioxidant and anti-inflammatory signalling. NRF2 is translocated for interaction with the antioxidant response element (ARE), inducing gene expression of antioxidants including superoxide dismutase (SOD), NAD(P)H, quinone oxioreductase-1 (NQO1), and heme oxygenase-1 (HO-1).

NRF2 is downregulated with progression of age and, in these circumstances or during states of chronic excess ROS production, NRF2-regulated cytoprotection and redox stability is hindered [32,33]. Several functions of NRF2 include the regulation of mitochondrial homeostasis, the regulation of antioxidant enzyme expression, prevention of cellular apoptosis, DNA repair, and transcriptional regulation [34,35,36]. An age-related reduction in NRF2 activity affects numerous other biochemical processes contributing to an increased ROS and subsequent ARHL [37].

In addition to mitochondria ROS, NAD(P)H oxidases (NOX) account for a moderate degree of ROS production. There are seven types of NOX enzymes in mammals in various locations in the body and with differing functions. One of their functions is shared between all NOX: oxidising O_2_ into O_2_^−^, which, either spontaneously or by SOD, can be converted into H_2_O_2_. Although the collective functions of NOX enzymes have not been entirely uncovered, it has been shown that this family has essential roles in host defence, the proliferation and differentiation of cells, cellular signalling, and apoptosis metabolism [38]. Similar to the mitochondrial redox regulation, NOX overactivation leads to oxidative stress and its associated cytotoxic damage. NOX3 is almost explicitly expressed within the inner ear, but its effects have been documented in emphysema development and insulin resistance in mice [38,39]. The p22^phox^ subunit is responsible for the activation of NOX 1–4. The reduction in NOX activity presented in p22^phox^ knockout mice attenuated hearing loss in addition to the preservation of cochlear morphology when compared to control wild-type mice. It has been posed that the targeting of NOX activity within the inner ear could be a potential pathway for therapeutic intervention of ARHL [40].

#### 4.1.2. Inflammation

Aging is accompanied by chronic inflammation, termed “inflammaging”, and dysregulation of the immune system attributable to oxidative stress, apoptosis activation, and mitochondrial dysfunction [41,42]. Chronic inflammation, consisting of persistent low-grade inflammation in tissues, is heavily linked to aging and an increased risk of cardiovascular diseases, diabetes, cancer, and senile dementia [41,43]. In addition to the persistent activation of the innate immune system, the chronic elevation of interleukin-6 (IL-6), Interleukin-1 beta (IL-1β), Interleukin-2 (IL-2) and tumour necrosis factor-alpha (TNF-α) are notable cytokines associated with auditory loss [24,42,44,45]. Chronic inflammation and ROS have been linked via the ROS activation of nuclear factor-kappa B (NF-κB). Long-term oxidative stress instigates chronic inflammation and proinflammatory cytokine production, which inadvertently increases ROS production. Another by-product of ROS is proinflammatory cytokine promotion, which in turn increases inflammation. This further increases ROS production and initiates a positive feedback effect that causes chronic damage [43,46]. The targeted inhibition of transcription factor NF-κB has been presented as a potential mediator of age-related inflammation [44,47]. Notably, NF-κB can be activated by ligand binding of TNF-α to the TNF-α receptor 1 (TNFR1) death ligand involved in the extrinsic apoptosis pathway. The result of this is proinflammatory cytokine synthesis ultimately increasing TNF-α, creating a positive feedback loop [48]. TNF-α has also been implicated in ARHL in context of both inflammaging and inner hair cell synaptic degeneration [42,49] The responsibilities of NF-κB are numerous and varied, functioning to regulate inflammatory responses, cell regeneration and proliferation, apoptotic signalling, and anti-apoptotic signalling. The states of oxidative stress that are triggered by or involve NF-κB can be both antiapoptotic or proapoptotic; thus, regulation requires contextually relevant understanding and targeting [50].

#### 4.1.3. Cellular Apoptosis

Apoptosis is a highly regulated process to induce cell death and has been shown as a major contributor to ARHL through hair cell death [51]. This process is regulated by genes and enzymes via the endoplasmic reticulum, intrinsic, and extrinsic pathways. Apoptosis pathways involve cysteine-aspartic protease (caspase) enzymes and is primarily regulated by Bcl-2 family proteins. In caspase-mediated apoptosis, the intrinsic and extrinsic pathways have unique initiator phases; but both share similar pathways in execution phase resulting in cell death via activation of caspase 3 and 7. The extrinsic pathway initiates via the binding of a death ligand to its concomitant death receptor, which belongs to the TNF superfamily. This stimulates a cascade of events leading to the death-inducing signalling complex (DISC) consequently activating caspase 8. The activation of caspase 8 stimulates the caspase 3 mediated execution phase. The intrinsic pathway is initiated via intracellular factors such as DNA damage, cytotoxicity, and ROS, which are sensed by BH3-only proteins. Bcl-2 family proteins, Bax and Bak, are stimulated by BH3-only proteins to create a mitochondrial outer membrane pore (MOMP), allowing the release of cytochrome C into the cytoplasm. The translocation of Bax and Bak are inhibited by antiapoptotic proteins of the Bcl-2 family including Bcl-2, preventing apoptosis. The cytochrome C binds to Apaf-1, activating caspase 9—the initiator of the execution phase. The executioner phase in these pathways involves the activation of caspase 3, 6 and 7, which function to destroy nuclear proteins, induce DNA and chromatin degradation, alter cytoskeleton arrangement, and disrupt cell division and communication. Ultimately, fragments of the cell are recognised and phagocytosed by epithelial cells and macrophages [52,53]. Both rat and mice models have been established for AHRL via d-galactose injection inducing H_2_O_2_-related oxidative stress-associated damage predominantly through mtDNA deletion and repeats, NOX overexpression, and uncoupling protein-2 (UCP2) promotion [54,55,56]. The expression of NOX3 in the stria vascularis, spiral ganglion, and organ of Corti, and the detection of cleaved caspase 3 cells was significantly increased in d-gal rats in comparison to the control rat model counterparts [57]. Previous research using d-Gal rats has demonstrated a significant increase in the NOX2 expression in the ventral cochlear nucleus and the NOX3 expression within the inner ear structures. These studies also highlighted mitochondrial morphological disruption and increased the cleaved caspase in the inner ear, inferring the involvement of the mitochondrial intrinsic pathway of apoptosis [54,57]. Research suggests that ROS act as p53 up-regulators, inducing continuous Bak activation, eventually leading to apoptosis in AHRL [58]. Similarly, in the C57BL/6J ARHL mouse model, Bak gene deletion prevented the apoptosis of hair cell and spiral ganglion neuronal cells [59].

#### 4.1.4. Autophagic Cell Protection

Autophagy is a regulatory mechanism triggered by the stimuli of environmental stress within a cell. The essential process denotes the containment, engulfment, and subsequent breakdown of degraded abnormal or damaged intracellular structures via the lysosome into more basic units (e.g., amino acids, nucleotides, fatty acids, etc.) for reuse [60]. Autophagy is regulated by rapamycin kinase mammalian target (mTOR). The inhibition of mTOR occurs via the p53 and adenosine 5-‘monophosphate activated protein kinase (AMPK) signalling pathway, increasing autophagy. The opposite occurs via the up-regulation of mTOR via MAPK and Akt pathway signalling, attenuating autophagy. During persistent oxidative stress, NRF2 reduces AMPK expression, suppressing extended autophagy and, thereby, preventing excessive destruction of cellular contents over time [61]. A reduction in NRF2 expression has been identified in ARHL, diminishing this protective mechanism and allowing a build-up of damaged and abnormal intercellular contents [62]. Protein synthesis occurs within the endoplasmic reticulum (ER). The ER evaluates synthesised proteins for abnormal assembly, subsequently destroying and recycling them when identified via either autophagy-lysosome or ubiquinone-proteasome systems [63]. Approximately, one-third of the proteins produced undergo this [60]. This process, termed the unfolded protein response (UPR), is a stress response, which may trigger apoptosis via caspase 12 release, c-Jun N-terminal kinases (JNK) expression, or upstream C/EBP homologous protein (CHOP) gene pathway activation when overwhelmed. In addition to this, chronic UPR activation and ER stress eventually inhibits autophagy, increasing the degree of misfolded and damaged proteins. Finally, chronic ER stress may trigger ROS production itself via NF-κB activation [53,64].

#### 4.1.5. Recent Findings

The linkage between diabetes mellitus (DM), dementia, and hearing loss is controversial; however, in the past decade, numerous studies have shown relationships between these pathologies, supporting the link [16,65,66,67,68,69,70]. Diabetes mellitus is characterised by chronic insulin insensitivity or deficiency, resulting in hyperglycemia and the degeneration of vasculature [71]. Dementia is also a chronic disorder of degeneration, presenting as cognitive decline and behavioural abnormalities. The underlying pathology includes, but is not limited to, neuronal loss in both the hippocampal and temporal regions, neurofibrillary tangles, vascular degeneration, and amyloid-beta plaque formation [70]. Metabolic dysregulation underlies all three diseases and the impact of this dysregulation increases with age, elucidating the categorization of these diseases to include geriatric syndrome [72]. The SAMP8 mouse model commonly used for ARHL is also used in contexts of diabetes and dementia due to the ubiquitous involvement of cellular senescence in all three pathologies [73,74,75]. Though DM and dementia are not the focus of this review, these developments show the potential for a greater understanding of ARHL, but also highlight potential future therapies with multi-targeted functional design.

## 5. Antioxidants

Antioxidants are molecules (either endogenous or exogenous) that serve to eliminate ROS or reduce the damage of ROS. The instigation of antioxidant release occurs in response to the presence of ROS within proximation to an extracellular matrix, cells, and tissues. The natural antioxidant defence of the body is via endogenous antioxidants, which, dependent on their function, are categorised as either enzymatic or non-enzymatic [76]. Both categories of endogenous antioxidants operate against differing ROS and within differing compartments of the cells, allowing the complementary mechanism of protection [43]. Glutathione reductase (GR), catalase (CAT), glutathione peroxidase (GPx), and SOD are enzymatic first-line defenders against ROS. Superoxide anions are catalysed into hydrogen peroxide via SOD, which is subsequently neutralised into a single water and oxygen molecule by CAT, GPx, or GR. Exogenous antioxidants are commonly found in diet and work symbiotically with endogenous antioxidants in restoration of cellular homeostasis [76].

Oxidative stress reduction requires a decrease in ROS or an increase in antioxidant activity. There are the following three mechanisms by which this can be achieved: the upregulation of endogenous antioxidants, introduction of exogenous antioxidants, and ROS-scavenger system promotion [77]. When addressing hearing loss prevention and management using antioxidants, it is imperative to be addressing multiple aspects of the highly multifaceted pathology. Several antioxidants have shown efficacy as a therapeutic intervention option in both noise-induced hearing loss and ototoxicity prevention but have not demonstrated similar benefits in ARHL. The pathogenesis of ARHL is still being elucidated; in addition, a practical and reliable delivery mechanism for therapeutic compounds to the inner ear is still required before any effective treatment can be pursued.

### Potential Antioxidants for Use in ARHL Treatment

Metformin is a hydrophilic, positively charged biguanide compound commonly used to treat type 2 DM as a sensitizer of insulin receptors and an antihyperglycemic agent. Its mechanism involves the stimulation of adenosine monophosphate-activated protein kinase (AMPK/ERK1/2), and through this mechanism has shown effects in anti-inflammatory, antiangiogenic, proapoptotic, and antioxidant contexts [78,79]. Metformin has shown to decrease ROS in long-term accumulation either via ROS scavenging or prevention of ROS production. In addition, metformin modulates inflammation and cellular degeneration and dysfunction associated with aging via NF-κB regulation [47]. Metformin was explored in the context of various aspects of hearing loss in d-galactose rats. Antioxidant activity (SOD, CAT and GSH) reduction increased in d-gal rats of 3 months, 9 months, and 15 months, respectively, when compared to age-matched counterparts in the control group. In the third group consisting of d-gal rats receiving metformin, the reduction in antioxidant activity seen in only d-gal group was attenuated to a significant degree. In addition to this, metformin abated mtDNA common deletions in both in vivo and in vitro groups, downregulated p53 expression and caspase 3 expression, prevented cellular apoptosis, decreased neurodegeneration associated with age, and finally, regulated UPR activity [80].

**Figure 1:**
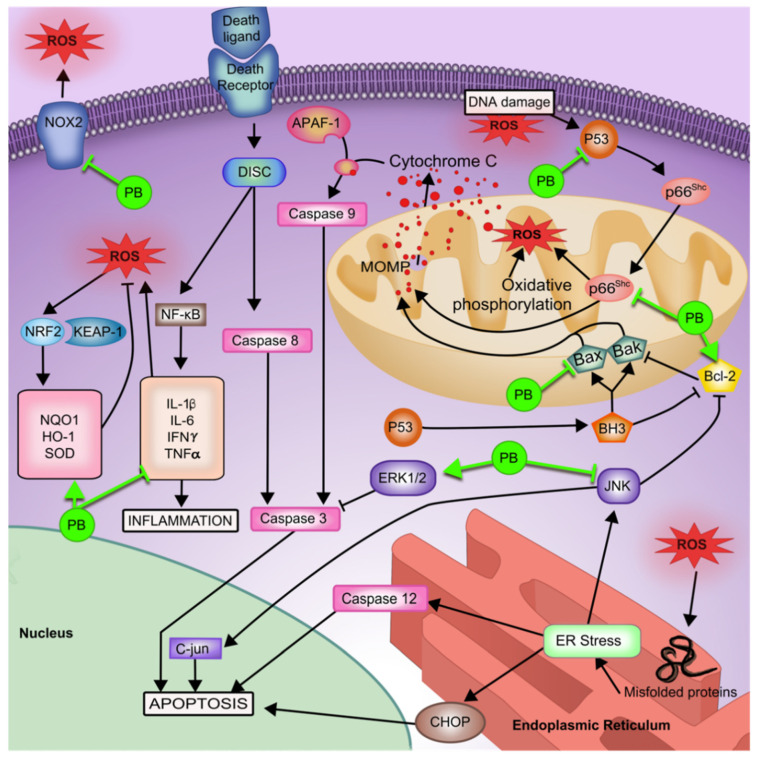
Schematic diagram of various cellular therapeutic effects of probucol in context of oxidative stress induced pathologies underlying ARHL. Abbreviations: PB, probucol; DNA, deoxyribonucleic acid; ROS, reactive oxygen species; NRF2, NF-E2-related factor 2; KEAP-1, Kelch-like ECH-associated protein 1; NQO1, NAD(P)H:quinone oxioreductase-1; HO-1, heme oxygenase-1; SOD, superoxide dismutase; DISC, death inducing signalling complex; IL-6, Interleukin-6; IL-1β, interleukin-1 beta; IFNγ, interferon-gamma; TNFα, tumour necrosis factor-alpha; NF-κB, nuclear factor-kappa B; BH3, BH3 only protein; MOMP, mitochondrial outer membrane pore; JNK, c-Jun N-terminal; ERK1/2, extracellular signal-related kinases 1 and 2; CHOP, C/EBP homologous protein; ER, endoplasmic reticulum.

Probucol is commonly used for its cholesterol-lowering capabilities, but it has also shown antioxidant and anti-inflammatory benefits. Its therapeutic antioxidant qualities have been noted in a diverse range of pathological contexts but are yet to be explored in the context of ARHL [81,82,83]. For example, probucol has been found to suppress ROS upregulated p66^Shc^ adaptor protein expression via NAD-dependent deacetylase sirtuin-1 (SIRT1) upregulation and also via the decrease and increase in upstream caspase 3 mediators, p-JNK, and activated extracellular signal-regulated kinases (p-ERK1/2), respectively [81,84]. The pathways activated by p66^Shc^ promotion can increase ROS production, reduce antioxidant production, and trigger cytochrome C-induced apoptosis [85]. In addition, probucol prevents apoptosis via Bax and p53 downregulation, and Bcl-2 upregulation [84,86]. Though the exact mechanism is not known, probucol shows antioxidative properties via the inhibition of NOX2 expression, which has been identified as a potential therapeutic target in ARHL [82]. The promotion of NRF2 activity has been noted in several studies in addition to its down-stream processes [83,87,88]. Probucol promotes recruitment of potent antioxidants NQO1, HO-1, and SOD [83,88,89]. The promotion of NQO1 exerts antioxidant effects by converting quinone to hydroquinone, effectively preventing quinone conversion into semiquinones via cytochrome P450 reductase. Semiquinones are subject to further a reaction with O_2_ due to its unstable nature, producing an ROS [36]. HO-1, on the other hand, is capable of oxidising prooxidant heme into carbon monoxide and, indirectly, bilirubin, which both possess cellular protective functions [90]. Given probucol’s up-regulation of NRF2 expression, it presents a viable option for potential benefits in ARHL prevention and management [33]. Therapeutic applications of probucol have been previously shown to reduce proinflammatory cytokines TNF-α, IL-1β, IL-6, IFN-γ, and increase the anti-inflammatory cytokine IL-10 [83,91,92,93]. Probucol provides extremely multifaceted mechanisms of assistance in ARHL, as summarised in Figure 1, but despite its effectiveness, probucol requires a well-developed method of drug delivery. Probucol is both low in bioavailability and highly lipophilic; and consequently, previous use of probucol has required increased doses to reach a therapeutic effect. Subsequently, heart arrythmias have been reported as a side effect due to the high inter-individual drug specificity [94].

These outlined antioxidants show potential as a therapeutic option in the antioxidant-based prevention of ARHL, either used alone or in combination. Regardless of the effectiveness that these compounds may provide, they need to be physically deliverable to the intended site, that is, the inner ear. This poses the following several challenges: the pharmacokinetics of the drug, and the inner ear locational difficulty. Microencapsulation and nanotechnology in drug delivery are known for their ability to improve delivery outcomes for unfavourable drug delivery profiles of pharmaceuticals, making them potential options for improving the delivery outcomes of metformin and probucol [95,96].

## 6. The Existing Barriers in Effective Treatment of Age-Related Hearing Loss

Held in the petrous bone—an extremely dense bone [97]—the inner ear provides a challenge when devising a targeted drug delivery method. Currently, a systemic administration of steroid through oral or intravenous routes is established as a treatment method for sudden sensorineural hearing loss and Meniere’s disease [98]. Systemic applications are problematic due to the omnipresent binding sites between both target and off-target cells. A consequence of this is the unfavourable off-target binding of the therapeutic agent and the subsequent adverse effects within the body [99]. Another concern with this delivery method is the sub-therapeutic levels of the drug reaching the target site due to the blood–perilymph barrier separating the inner ear from systemic circulation [99,100]. Larger doses of the treating drug are required for effective therapeutic dosage, further increasing the risk of systemic side effects [101]. Local delivery to the inner ear is being explored as a promising route of drug delivery, thereby, eliminating the risks associated with systemic drug exposure and decreasing the required dose for therapeutic effect [100].

The round window membrane (RWM) has traditionally been understood as the main route of transport separating the middle and inner ear compartments. It consists of an inner and outer epithelial layer with a middle layer of connective tissue, combining to be an average thickness of 70 µm. Contained within the connective tissue layer are elastic fibres, collagen, and fibroblasts in addition to lymph and blood vessels [102]. The permeability of the RWM depends on the size, surface charge, hydrophilicity, RWM thickness, and total contact time with the RWM [103,104,105,106,107,108,109,110,111]. Clearance of the middle ear via the Eustachian tube is another concept to consider when designing an inner ear drug delivery system. This consistent elimination of the contents of the middle ear can result in the necessity for repeated injections to achieve therapeutic levels, prompting research efforts into controlled drug release and the retainment of drug carriers in the middle ear [95,112,113,114,115].

## 7. Local Drug Delivery of Antioxidants to Inner Ear

Research on and the application of drug delivery systems to the inner ear is shifting to local delivery systems aiming to avoid the blood–perilymph barrier. In addition, this form of delivery allows drugs either low in bioavailability and/or known to induce systemic side effects, such as metformin and probucol, to be delivered more effectively and safely. As previously mentioned, probucol has historically caused cardiac arrythmias in patients due to its inter-individual variability in oral bioavailability. The combination of both the low bioavailability of metformin and the challenge of crossing the blood–perilymph barrier makes local delivery a more appealing delivery system moving forward in inner ear delivery [79,94,100]. Local drug delivery can be achieved via transtympanic, intracochlear, or intratympanic delivery (ITD) [98]. Transtympanic delivery involves application of the pharmaceutical to the tympanic membrane for permeation into the middle ear. The non-invasive nature of this application makes it favourable for patient comfort, but this is at the expense of the bioavailability of the drug at the target site. Intracochlear delivery to the inner ear involves the delivery of drugs directly into the cochlea through invasive surgery. Bioavailability is a major benefit to this technique, but it requires a procedure with specialised staff in a hospital. The risks of acoustic trauma are increased via disruption of the delicate fluid balance within the cochlea and the subsequent sheer stress of hair cell, toxicity due to high drug concentration, or via introduction of a pathogen into the inner ear. The methodology of ITD involves the delivery of the drug directly into the middle ear for diffusion into the inner ear, bypassing the tympanic membrane and reducing the acoustic trauma risks associated with intracochlear delivery. The success of ITD delivery depends heavily on transport across the RWM [95].

The thickness of the RWM is variable between individuals to some degree but is significantly increased in those with otitis media, thus decreasing its permeability [103,104,105]. With regard to size, smaller particles show greater permeability to the RWM than larger particles in a study comparing three nanocarriers of varying size. The largest nanocarrier (240 nm) demonstrated the lowest permeability and the smallest nanocarrier (90 nm) demonstrated the highest permeability [106]). Previous studies have shown an increase in RWM permeability resulting from positively charged molecules [107,108,109]. A study by Liu et al. analysed glycerol monooleate-based nanoparticles of varied charges and their transport characteristics regarding L929 cells, the RWM, and general cochlear distribution. It was concluded that the nanoparticles modified to have positive surface charge increased the permeability of the RWM and had an increased uptake by the L929 cells in addition to a greater overall cochlear biodistribution of the drug [108]. Comparatively, the RWM has shown higher affinity for the permeation of hydrophobic drugs compared to hydrophilic drugs [110]. The influence of change in concentration was explored when comparing a 190 mOsm and 620 mOsm trimethylphenylammonium (TMPA) in artificial perilymph solution. Permeability was increased by a factor of 2–3 for the higher drug concentration [111]. This behaviour has also been presented in Poly (lactic-*co*-glycolic acid) (PLGA) nanoparticles [116]. An increased contact duration of a solution with the RWM subsequently increases its permeability, as shown with PLGA nanoparticles [116] and TMPA solution [111].

**Figure 2:**
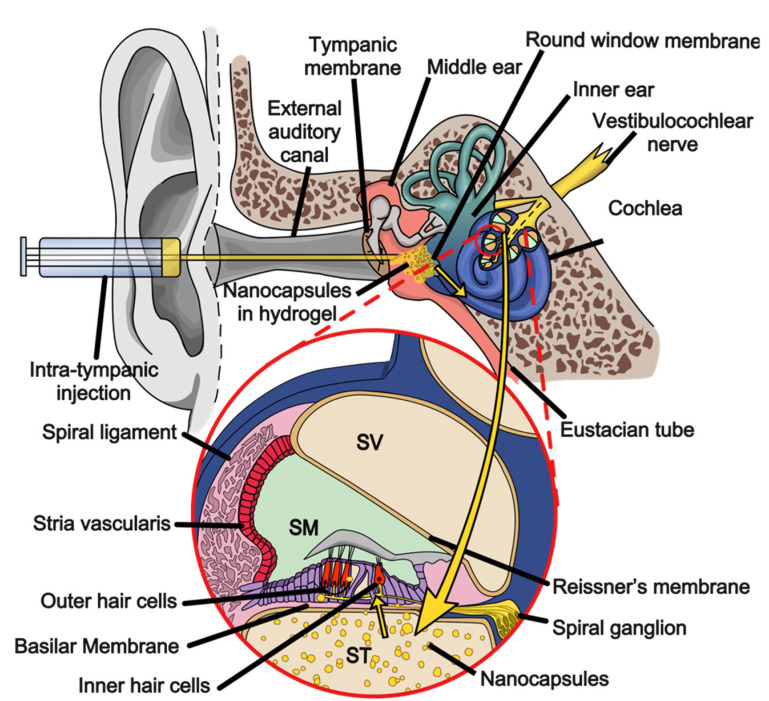
Illustration denoting intratympanic drug delivery of hydrogel nanocapsules into the cochlea. Injection bypasses transtympanic membrane into middle ear cavity and deposits drug in contact with round window membrane. (SV = Scala vestibuli, SM = Scala media, ST = Scala tympani).

In consideration to the barriers presented by diffusion across the RWM and Eustachian tube clearance rates, devices and modifications have been formulated to mitigate these challenges, improving ITD delivery [95,98,117,118,119]. Increasing the viscosity or mucosal adherence properties of an applied drug reduces Eustachian tube clearance, as seen in hydrogels [120,121,122]. Gelatinous sponges can be surgically placed within the middle ear for sustained drug release; [123,124,125] and finally, nanoparticle delivery systems are highly modifiable delivery systems allowing tailored approached to the improvement of RWM permeability [95,126,127]. Combinations of these methods are also explored, such as nanoparticle delivery in conjunction with hydrogels, gelatinous sponges or foams, as illustrated in Figure 2 [121,128].

## 8. Nano and Micro Drug Carriers

Both microcapsules and nanoparticles are characterised by their size [129,130,131,132]. Their physiochemical characteristics and properties, such as shape, surface composition, excipients, and surface charge, are highly customizable, allowing for alleviation of unfavourable drug properties such as poor solubility, ineffective diffusion, degradation, and reduced half-life [126,133,134]. For a nanocarrier to be suitable for ITD, the following requirements need to be met: it must be able to load a sufficient amount of drug, resistant to clearance via Eustachian tube, transportable across the RWM, protective of delicate drugs, and able to maintain a prolonged RWM contact [95,126]. When designing a nanocarrier delivery system, the excipients and method of formulation for the carrier determine the physiochemical characteristics of the finalised capsule [126,135,136,137,138]. Several reviews have summarised the benefits of nanocarriers in applications to inner ear delivery, highlighting improvements in biodistributions and availability, RWM transportability, otoprotective effects, and the reduction in the targeted effect in the outer hair cells and stria vascularis [95,113,139]. The prospect of microencapsulation for inner ear drug delivery has not yet been explored, but due to its larger size, would not seem to be a viable option for RWM transport. However, microcapsules still maintain potential for ITD through sustained release if they are immobilised whilst in contact with the RWM for an extended duration of time [112,114]. Nanoencapsulation and microencapsulation of low-bioavailable drugs is a favourable method for improving bioavailability and transport characteristics, especially when using bile acids as an excipient in the encapsulation [135].

## 9. Bile Acids

Primary bile acids are endogenous molecules formed in the liver via several enzymatic alterations of the precursor, cholesterol [140,141]. The subsequent conjugation with taurine or glycine forms bile salts that possess reduced membranolytic and cytotoxic properties than their primary bile acid counterparts. These conjugates are stored within the gallbladder for postprandial release into the lumen of the small intestine. The bile salts released into the lumen aid in digestion and the subsequent absorption of lipophilic molecules via mixed micelle formation [142]. Within the large intestine (ileum and colon), unused bile salts are deconjugated by local microbiota and enzymatic action into secondary bile acids for reabsorption. The reabsorption of bile acids occurs at 95% efficacy, transporting bile acids back to the liver in a process called enterohepatic circulation. This process allows a consistent bile acid pool to be maintained for lipid digestion, without the requirement for increased production and higher, toxic levels of bile acids. In addition to digestive functions, bile acids have shown roles in endocrine signalling, the prevention of gall and kidney stone production, microbiota maintenance, and both local and immune functions [143]. In the recent literature, bile acids have shown, in addition to exerting therapeutic effect themselves, remarkable benefits for improving nanoencapsulation and microencapsulation in drug delivery outcomes [3,144]. The structure of bile acids consists of both hydrophobic and hydrophilic structural components. This results in bile acids possessing amphipathic characteristics that facilitate permeation enhancing effects across membranes due to detergent-like action. This enables the solubilisation of membranes and the improved permeability of lipophilic molecules possessing low bioavailability and high interindividual variation in pharmacokinetics, such as probucol [145]. The safety of bile acids when incorporated as an excipient in drug delivery has previously been questioned. Apoptosis, cytotoxicity, and ROS production are concerns when exploring physiological effects of pharmacologically introduced bile acids; however, these effects are predominantly associated with concentrations exceeding physiological parameters [3]. Several bile acids have been used in the encapsulation of probucol with aims of improving targeted drug delivery. Numerous studies of this nature have shown significant benefits demonstrating bile acids as attractive excipients in the formation of nanocarriers for drug delivery [92,131,146,147,148,149,150,151,152,153,154,155,156]. When considering bile acids in the context of inner ear drug delivery, bile acids show promise in improving sustained and controlled drug release to the inner ear.

### 9.1. Bile Acid Synthesis, Metabolism and Microbial Crosstalk

Synthesis of primary bile acids involves over 17 enzymes via the following two potential pathways: the classical pathway and the alternative pathway. The classical pathway accounts for an excess of 90% of primary bile acid synthesis and begins with cholesterol 7α hydroxylase (CYP7A1)—mediated hydroxylation of cholesterol’s C7 sterol ring. From this point, the determination of cholic acid (CA) or chenodeoxycholic acid (CDCA) production is established by the enzymatic activity of either 12α hydroxylase (CY8B1) or sterol 27-hydroxylase (CYP27), respectively. The alternative pathway mainly synthesizes CDCA via CYP27 and the subsequent oxysterol 7-α hydroxylase (CYP7B1)-mediated transformation of cholesterol. Prior to release, enzymes acid-CoA:amino acid N-acyltransferase and bile acid-CoA synthase conjugate CA and CDCA with either glycine or taurine, improving their pharmacokinetics properties for digestive function [144]. The conjugation of primary bile acids increases the hydrophilicity and side chain acidity in addition to lowering the approximate molecular pKa of the primary bile acids from 5.0 to < 2 for taurine conjugates and 3.9 for glycine conjugates. The benefits of this include the increased hydrophilicity and ionization, and the associated reduction in the precipitation in acidic solutions, such as in the stomach [3].

Conjugated bile acids are released into the duodenum post food consumption, where the amphipathic structure and low pKa of the conjugated bile acids allows micelle formation to aid lipid and lipid-based vitamin digestion and absorption. A further transformation to the bile acids occurs within the colon. The local bacteria capable of expressing bile salt hydrolase deconjugate the glycine or taurine from the bile acids, then the bacterial expression of bile acid 7α-dehydroxylase removes the 7α hydroxyl group from the deconjugated primary bile acids. The dehydroxylation of CA forms deoxycholic acid (DCA) and the dehydroxylation of CDCA forms lithocholic acid (LCA), both of which are more lipophilic than their primary bile acid counterparts. The lipophilicity of these bile acids is also associated with an increased membranolytic and toxic effect [157]. Throughout this process, conjugated bile acids are also being actively reabsorbed via apical sodium-dependent bile salt transporter for transport via the portal circulation back to the liver for reuse. Deconjugated bile acids are also capable of passively diffusing through the apical colon for reuse. Bile acid ligand biding to the hepatic farsenoid X receptor (FXR) activates a pathway of signalling resulting in negative feedback to both CYP7A1 enzyme-expressing genes, preventing their transcription and, thus, preventing the synthesis of bile acid via cholesterol catabolism. This feedback system prevents the overproduction of bile acids that may reach toxic levels [144,158].

### 9.2. Bile Acids in Drug Delivery

The secondary bile acid, DCA, is known for its ability to increase membrane permeability in various physiological settings [159,160,161,162]. A study by Lalić-Popović et al. [159] investigated the effects of DCA on the permeability of gliclazide across the blood–brain barrier in healthy and diabetic rats. The addition of DCA to gliclazide promoted the transport of gliclazide across the blood–brain barrier to a factor of three in healthy rats and a factor of four in diabetic rats [159]. Several studies have shown that the addition of DCA to drug carriers improves the mechanical and osmotic stability of drug carriers, improved controlled release delivery outcomes, and chemical stability with probucol addition [93,130,132,136,148,154,163,164,165,166,167,168,169,170,171,172,173,174,175,176,177,178,179]

Characterised as both a secondary and a tertiary bile acid [176,180,181], ursodeoxycholic acid (UDCA) has demonstrated antioxidant effects within the cochlea through the attenuation of miR-34a expression. Using C57BL/6 ARHL mice models and HEI-OC1 cell lines, Pang et al. analysed the relationships between miR-34a over-expression, regulatory autophagy activity, and the effects of UDCA within this context. Several key findings were revealed. Firstly, C57BL/6 mice models were effective models of ARHL. Secondly, miR-34a over-expression was significantly increased in the C57BL/6 model in comparison to the 1- and 3-month-old control mice. Thirdly, the transfection of HEI-OC1 cells suppresses regulatory autophagy, subsequently inducing cell death via the downregulation of ATGA9A. Finally, UDCA treatment significantly downregulated miR-34 expression and significantly reduced the cell death of miR-34-transfected cells [182]. UDCA incorporation into nano and micro capsule for drug delivery has demonstrated improved permeability [147] cytoprotective effects [183], and antioxidant effects [92,135,146,184,185,186,187,188].

Tauroursodeoxycholic acid (TUDCA) is formed by the conjugation of UDCA with taurine and has been shown to possess neuroprotective [189], hepatoprotective, and otoprotective effects [190]. TUDCA has been shown to attenuate the activation of all three pathways of the UPR response in small intestine Mode-K cells, inhibiting GRP78 gene expression [191]. In a murine model of nonsydromic autosomal recessive deafness induced via Cdh23^erl/erl^ mutation, injections of TUDCA effectively ameliorated OHC apoptosis when compared to those injected with a phosphate buffer solution. The mechanism by which this occurs needs to be further explored, but the otoprotective effects were evident [190]. The incorporation of TUDCA in drug delivery has also demonstrated the permeating enhancing effects expected of bile acids [192]

LCA is produced in the colon via bacterial deconjugation [144]. In Caco-2 intestinal epithelial cells, this secondary bile acid prevented TNF-α-induced epithelial damage. There was evidence of partial mediation via the downregulation of SIRT1-NRF2 pathways and the upregulation of NF-κB significantly attenuating ROS production and inflammation. This was achieved through the agonistic effects of LCA on the vitamin D receptor in the gastrointestinal tract [193]. The relationship between vitamin D and hearing loss has previously been explored [194]. Vitamin D receptor knockout mice show spiral ganglion degeneration, hair cell loss, increased cleaved caspase 3 biomarkers, and expression of the vitamin D receptor in the cochlea itself [195]. High levels of LCA have shown toxic effects in the body, specifically hepatotoxicity. Conversely, the incorporation of LCA in drug delivery systems has shown cytoprotective effects when encapsulating NIT-1 pancreas β-cells [92].

## 10. Conclusions

The increasing global prevalence of ARHL has influenced the focus of research with a growing need for answers and action. Identified via the utilisation of a search strategy implementing several databases, recent discoveries have further unravelled the complex and multifaceted pathogenesis of ARHL. In conjunction with this, the potential suitability of bile acid-based drug delivery mechanisms was explored in the context of antioxidant-based therapies as treatment for this disease. With the understanding of the pathogenesis of ARHL growing over time, the connection it holds with oxidative stress and chronic inflammation is becoming more apparent. These findings allow more efficacious pharmacologic interventions to be optioned as potential therapies. Several of the target molecules suggested in the recent literature for therapeutic intervention relate to the therapeutic mechanisms of action of common medications such as probucol and metformin; however, the need for a multifaceted approach to treatment still requires a suitable drug delivery system capable of delivering the required pharmaceutics to the affected areas. Systemic delivery of treatment drugs is the optimal choice when approaching from a patient compliance perspective, but, unfortunately, this is not a likely solution given the current state of biotechnology and understanding in this field. Thus, trends in inner ear drug delivery research show a heightened focus on local rather than systemic delivery, attempting to achieve sufficient therapeutic dosing within the cochlea whilst avoiding systemic side effects. Recent findings in bile acid incorporation in nano and micro drug delivery systems provide evidence supporting bile acids as excipients for several potential platforms for drug delivery into the inner ear. In addition, new research is emerging in the use of bile acids, such as TUDCA, as the therapeutic agent itself. Future research may benefit from exploring the therapeutic effects of bile acids in ARHL and how they may be utilised in improving drug delivery outcomes when aiming to overcome the anatomical barriers associated with inner ear drug delivery.
